# All-Optical Parametric-Resonance Magnetometer Based on ^4^He Atomic Alignment

**DOI:** 10.3390/s22114184

**Published:** 2022-05-31

**Authors:** Bowen Wang, Xiang Peng, Haidong Wang, Wei Xiao, Hong Guo

**Affiliations:** State Key Laboratory of Advanced Optical Communication Systems and Networks, School of Electronics, Center for Quantum Information Technology, Peking University, Beijing 100871, China; bowen-wang@pku.edu.cn (B.W.); whd@pku.edu.cn (H.W.); xiao_wei@pku.edu.cn (W.X.); hongguo@pku.edu.cn (H.G.)

**Keywords:** parametric-resonance magnetometer, helium atom, light shift

## Abstract

Parametric-resonance magnetometer is a high-sensitivity quantum sensor characterized by applying the non-resonant radio-frequency (RF) fields to the atomic ensemble. The RF fields lead to crosstalk in the multi-sensor design, thus disturbing the magnetic-field measurement results. We propose an optically modulated alignment-based ${}^{4}$He parametric-resonance magnetometer. By using the fictitious field generated by the modulated light shift, parametric resonance is realized, and crosstalk caused by the magnetic RF field is prevented. The relative intensity noise of the lasers is suppressed to optimize the sensitivity of the magnetometer. Our magnetometer experimentally demonstrates a magnetic-field noise floor of 130 $\;\mathrm{fT}/{\mathrm{Hz}}^{1/2}$ in both open- and closed-loop operations and has the potential to reach 70 $\;\mathrm{fT}/{\mathrm{Hz}}^{1/2}$ when compared with the optimized magnetic RF scheme. It provides near-zero magnetic-field measurements with a 2 kHz bandwidth at room temperature, which is useful for high-bandwidth measurements in biomagnetic applications.

## 1. Introduction

Parametric resonance in atom-photon interactions is interpreted as the response of atoms dressed by non-resonant “radio-frequency (RF) photons” to variations in external magnetic fields [1,2,3,4,5]. This effect was used for the first time in ${}^{87}$Rb atoms to achieve the high-sensitivity detection of a near-zero magnetic field produced by oriented nuclei of ${}^{3}$He atoms [6,7]. In the 1970s, the parametric-resonance magnetometer (PRM) was implemented in ${}^{4}$He atoms to measure interplanetary fields [8,9] and geomagnetic fields [10]. Integrated commercial PRMs designed by QuSpin Inc. [11] and Twinleaf LLC [12] have been successfully applied to measure fields from humans [13], plants [14], and pre-polarized nuclear magnetic resonance samples [15]. These types of orientation-based PRMs pumped with circularly polarized light have been extensively studied, and the atomic ensembles can be applied with single [16,17], double [18,19], or even triple [20] non-resonant transverse RF fields for different applications. In addition, there is a longitudinal geometry of the PRM in which the non-resonant RF field is parallel to the optical pumping direction, and an additional probe light is required [21,22,23]. The tradeoff between transverse and longitudinal geometries has also been discussed in a related study [24]. In addition, the manipulation of Landé *g*-factor due to the “RF photons” dressing in PRM has attracted much attention [25,26,27,28], and the parametric modulation can be introduced into other types of optically pumped magnetometers, such as the combination of PRM and nonlinear magneto-optical rotation (NMOR) [29], magneto-optical double resonance (MODR) [30], or spin-exchange relaxation free (SERF) magnetometers [31].

The macroscopic magnetic moment of the atomic ensemble in the above-mentioned orientation-based PRMs may disturb the sensor, and it is difficult to adjust the pumping direction unless the direction of light propagation is changed. Therefore, alignment-based PRM pumped with linearly polarized light has been rapidly developed, especially for ${}^{4}$He atoms [32,33]. An essential advantage of ${}^{4}$He atoms is that neither high-temperature heating (such as alkali metal PRM) nor cryogenic cooling (such as superconducting quantum interference devices [34]) is required. In biomedical applications, alignment-based ${}^{4}$He PRM reported in magnetocardiography [35] and magnetoencephalography [36] can be operated at room temperature to enable the sensor to be close to the skin surface without causing discomfort. For practicality, the alignment-based ${}^{4}$He PRM has also been designed to work in an unshielded environment [37] or can be integrated as a multi-sensor array [38]. It is worth mentioning that PRM pumped with elliptically polarized light is also feasible [39], which combines atomic orientation and alignment.

A challenging problem in multi-sensor design is that the RF field induces crosstalk between adjacent sensors. To solve this problem, the possibility of using a time-dependent fictitious field to replace the magnetic RF field was first proposed in an experimental study of light shift [40]. With the help of vector light shift, the interaction of detuned circularly polarized light with the atomic ensemble can generate an effective fictitious field collinear with the pumping axis. In recent years, there has been renewed interest in the operation of optically pumped magnetometers using fictitious fields, such as all-optical NMOR [41], MODR [42,43,44], and SERF magnetometers [45]. Another advantage of introducing the fictitious field is that the magnetic shield is not perturbed during the experiments because only the atomic ensemble that interacts with the light-shift beam can sense the fictitious field. Considering the elimination of the adverse effects of the magnetic RF field, a promising alignment-based ${}^{4}$He PRM merits an all-optical design.

In this study, we propose and experimentally demonstrate an optically modulated alignment-based ${}^{4}$He PRM driven by a fictitious field. Open- and closed-loop operations of the parametric resonance were performed to measure the quasi-static near-zero magnetic field at room temperature. By using the modulated light shift, the magnetic RF field, which causes crosstalk in the multi-sensor design, is prevented. We stabilized the output power of the lasers to optimize the sensitivity of the magnetometer. Compared with the optimized magnetic RF scheme using the same apparatus, the magnetic-field sensitivity of the proposed PRM is expected to be further improved, and three-axis detection can be achieved by adding another light-shift beam. Our PRM is useful for high-sensitivity measurements in biomagnetic applications.

## 2. Principle and Theory

**Figure 1:**
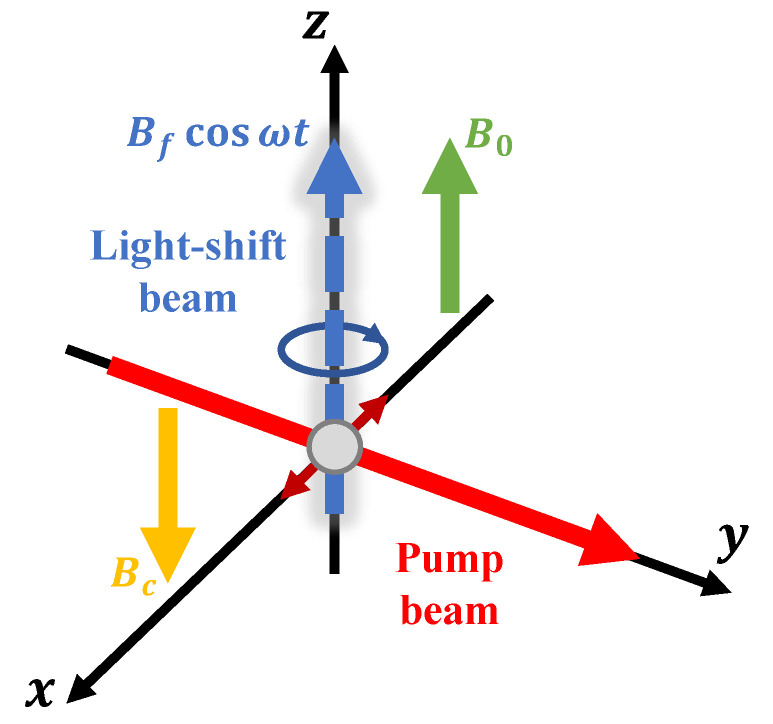
Field geometry of the optically modulated alignment-based ${}^{4}$He parametric-resonance magnetometer operated in the closed-loop mode. $B_{0}$, quasi-static field to be measured; $B_{f}$, fictitious field; $B_{c}$, compensating field. The gray circle at the center of the coordinate system represents ${}^{4}$He atomic ensemble. In the open-loop mode, the $B_{c}$ field is removed.

The field geometry of the proposed PRM is illustrated in Figure 1. A linearly *x*-polarized pump beam interacts with an ensemble of F=1 metastable ${}^{4}$He atoms at $D_{0}$ ($2^{\;3\;}S_{1}\to 2^{\;3\;}P_{0}$) transition and forms alignment atomic polarization. Parametric resonance under a quasi-static magnetic field $B_{0}$ is induced by an oscillating fictitious field $B_{f}\cos\left(\omega t\right)$ along the *z*-axis with amplitude $B_{f}$ and frequency ω, generated by the interaction of an intensity-modulated circularly polarized detuned beam (also called a light-shift beam) with atoms. Changes in the $B_{0}$ field affect the evolution of the polarized atomic ensemble, which alters the absorption of the pump beam transmitted through the ${}^{4}$He gas cell. Therefore, the connection between the magnetic field to be measured and the photodetection signal is established. In the open-loop mode, we convert the calibrated response voltage to the magnetic field to be measured. While in the closed-loop mode, a feedback coil is used to generate a compensating field $B_{c}$ to lock the magnetic field around the atomic cell at the zero fields, and the value of $B_{0}$ field can then be read from the coil current in real time.

The fictitious field originates from energy variations in Zeeman sublevels caused by the vector light shift. By taking the effective operator formalism [46], the effective light-shift Hamiltonian of metastable ${}^{4}$He considering the $D_{0}$ transition can be written as [47]

(1) $$\delta \hat{\varepsilon }=\frac{\tau^{2}E_{0}^{2}d_{0}^{2}\hat{\Phi }}{12\hbar }\frac{\Omega -\Omega_{0}}{1+\tau^{2}{\left(\Omega -\Omega_{0}\right)}^{2}},$$

where *ℏ* is the reduced Planck constant and τ is the lifetime of the excited state $2^{\;3\;}P_{0}$, $\Omega_{0}$ is the $D_{0}$ transition frequency, and $E_{0}$ and Ω are the electric-field amplitude and optical frequency of the light-shift beam, respectively. Further, $d_{0}=2.5312ea_{0}$, *e* is the electron charge, and $a_{0}$ is the Bohr radius. $\hat{\Phi }$ is a 3×3 matrix related to the polarization of the light-shift beam for electric dipole interactions, which can be expanded into irreducible tensor operators [48]:

(2) $$\hat{\Phi }=\sum_{\kappa =0}^{2}\sum_{q=-\kappa }^{\kappa }{\left(-1\right)}^{q}\langle {\hat{T}}_{-q}^{\left(\kappa \right)}\rangle {\hat{T}}_{q}^{\left(\kappa \right)}=\sum_{\kappa =0}^{2}\sum_{q=-\kappa }^{\kappa }\Phi_{q}^{\left(\kappa \right)}{\hat{T}}_{q}^{\left(\kappa \right)},$$

where ${\hat{T}}_{q}^{\left(\kappa \right)}$ is the *q*-th (q=−κ,−κ+1,⋯,κ−1,κ) component of the rank-κ (κ=0,1,2) irreducible tensor operator, and $\Phi_{q}^{\left(\kappa \right)}$ is the corresponding coefficient whose detailed expression in the case of metastable ${}^{4}$He can be verified in a previous study [49]. In Equation (1) with expanded $\hat{\Phi }$, only the term containing ${\hat{T}}_{0}^{\left(1\right)}$ is related to the vector light shift with the quantization axis (*z*-axis) along the direction of the light-shift beam. Note that ${\hat{T}}_{0}^{\left(1\right)}=\hat{J_{z}}/\sqrt{2}$ in spin-1 space, where $\hat{J_{z}}$ is the angular momentum component. By making the vector-light-shift term equivalent to the energy displacement caused by the magnetic field, that is, $\delta \hat{\varepsilon }\left(\kappa =1,q=0\right)=g_{J}\mu_{B}B_{f}\hat{J_{z}}$, we obtain the analytical expression of the fictitious field in the $D_{0}$ transition:

(3) $$B_{f}=\frac{\sqrt{2}\tau^{2}E_{0}^{2}d_{0}^{2}\Phi_{0}^{\left(1\right)}}{24g_{J}\mu_{B}\hbar }\frac{\Omega -\Omega_{0}}{1+\tau^{2}{\left(\Omega -\Omega_{0}\right)}^{2}},$$

where $g_{J}$ is the Landé *g*-factor and $\mu_{B}$ is Bohr magneton.

In alignment-based zero-field ${}^{4}$He parametric resonance, the evolution of the pump-beam absorption *S* (i.e., photodetection signal) at the fictitious-field frequency ω is [32]

(4) $$S\propto J_{0}\left(\frac{2\gamma B_{f}}{\omega }\right)J_{1}\left(\frac{2\gamma B_{f}}{\omega }\right)\frac{-\Gamma_{p}\gamma B_{0}}{{\left(\Gamma_{p}+\Gamma_{e}\right)}^{2}+4\gamma^{2}B_{0}^{2}}\sin\left(\omega t\right),$$

where $J_{n}$ is the *n*th-order Bessel function of the first kind, γ is the gyromagnetic ratio of metastable ${}^{4}$He, $\Gamma_{e}$ is the metastable collision relaxation rate, and $\Gamma_{p}$ is the optical-pumping relaxation rate. The amplitude of Equation (4) exhibits a Lorentzian dispersive profile centered at $B_{0}=0$.

## 3. Experiment and Results

### 3.1. Experimental Setup

**Figure 2:**
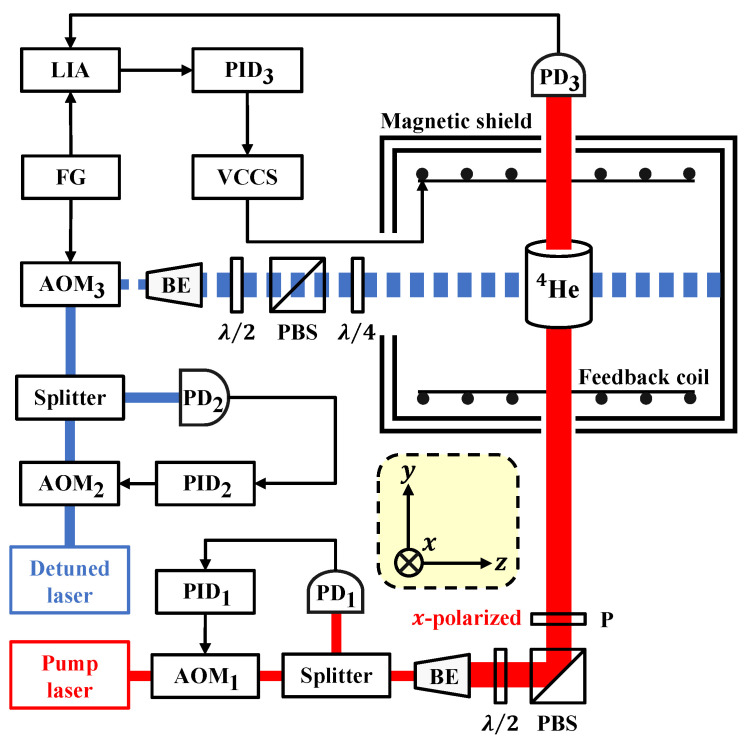
Schematic diagram of the optically modulated alignment-based ${}^{4}$He parametric-resonance magnetometer operated in the closed-loop mode. AOM, acousto-optic modulator; PD, photodiode; BE, beam expander; PBS, polarization beam splitter; P, polarizer; λ/2, half-wave plate; λ/4, quarter-wave plate; PID, proportional-integral-derivative controller; FG, function generator; LIA, lock-in amplifier; VCCS, voltage-controlled current source. In the open-loop mode, the feedback coil, VCCS, and ${\mathrm{PID}}_{3}$ are removed.

We operated the proposed PRM as shown in Figure 2 at room temperature. The pump beam emitted by a 1083.206 nm frequency-stabilized fiber laser (NKT Koheras BOOSTIK Y10) resonating with the $D_{0}$ transition of metastable ${}^{4}$He is incident onto an intensity-noise-suppression subsystem composed of a commercial acousto-optic modulator, self-designed proportional-integral-derivative controller, customized photodiode, and beam splitter. The pump beam reflected from the polarization beam splitter is *x*-polarized and passes through a polarizer to enhance its extinction ratio. The light-shift beam emitted from another fiber laser (NKT Koheras BASIK Y10 with an OEM amplifier), whose working wavelength is slightly detuned from the $D_{0}$ transition, is also subjected to intensity-noise suppression. Then, the light-shift beam is sinusoidally intensity-modulated by the third acousto-optic modulator (${\mathrm{AOM}}_{3}$ in Figure 2) at a frequency of ω=2π×1.33kHz and is converted to circular polarization using a quarter-wave plate. The waist diameter of both light beams is expanded to 12 mm ($1/e^{2}$) by the beam expanders. The half-wave plates are used to optimize light intensities with the polarization beam splitters next to them. The RF drivers (AA Opto-electronic MODA110-B4-34) of the three acousto-optic modulators (AA Opto-electronic MT110-IR25-3FIO) are not shown in the schematic diagram.

The intensity of the pump beam is 0.5 mW, and the mean intensity of the sinusoidally modulated light-shift beam is 10 mW. The pump beam and light-shift beam propagate along the longitudinal and orthogonal axes of the cylindrical gas cell placed in a seven-layer μ-metal magnetic shield. The 50-mm-diameter, 70-mm-long gas cell filled with 76 Pa of ${}^{4}$He pressure is excited by an electrodeless discharge at 39.6 MHz frequency absorbing 100 mW power, which populates the metastable state $2^{\;3\;}S_{1}$. The absorption of the pump beam after the ${}^{4}$He gas cell is detected using the third photodiode (${\mathrm{PD}}_{3}$ in Figure 2), and the magnetic-field response by which we can achieve open- and closed-loop operations separately is obtained by demodulating the photodiode signal at frequency ω with a lock-in amplifier (Zurich Instruments HF2LI) controlled by LabOne software. Note that the function generator is integrated into the lock-in amplifier.

The proportional-integral-derivative parameters of the two intensity-noise-suppression subsystems were tuned using the standard Ziegler–Nichols step response method [50]. After the optimal parameters were determined, the relative intensity noise floor of the pump beam was optimized from −122 dBc/Hz to −132 dBc/Hz, and that of the light-shift beam was optimized from −132 dBc/Hz to −138 dBc/Hz.

### 3.2. Fictitious Field

As described in Equation (3), the amplitude of the fictitious field is determined by the wavelength and intensity $I_{0}\propto E_{0}^{2}$ of the light-shift beam. To minimize the complexity when considering the fictitious field from the $D_{1}$ and $D_{2}$ transitions, we choose a wavelength that is blue-detuned from the $D_{0}$ transition for the light-shift beam, which is far away from the $D_{1}$ and $D_{2}$ transitions. According to the slope of the magnetic-field response, the wavelength of the light-shift beam is set to an optimal value of 1083.195 nm. In addition, the wavelength of the light-shift beam is monitored using a wavelength meter (Bristol Instruments 771A-NIR) before entering the ${}^{4}$He gas cell.

**Figure 3:**
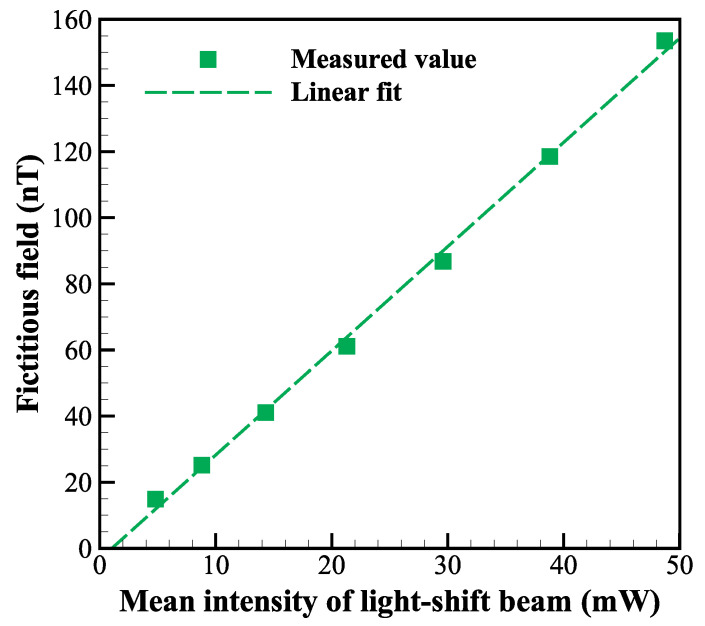
The relationship between the mean intensity of the light-shift beam with a wavelength of 1083.195 nm and the magnitude of the light-shift fictitious field. The square symbols are the measured values, and the dashed line is the corresponding linear fit.

After the wavelength was fixed, we studied the relationship between the optical power of the light-shift beam and the magnitude of the fictitious field to give a definite fictitious-field coefficient of our scheme. During calibration, we temporarily removed the intensity-noise-suppression subsystem on the optical path of the light-shift beam to investigate a wider range of optical power. The function generator gives a parametric-resonance modulation with an amplitude of 1 V to the RF driver (input range is 0 to 5 V) of ${\mathrm{AOM}}_{3}$, and the modulation offset is varied from 1 to 4 V. We recorded the displacements of the magnetic-field response due to the variations of the fictitious-field offset as a function of the mean intensity of the light-shift beam, and the results are shown in Figure 3.

The mean intensity is recorded by Thorlabs PM100D with standard power sensor S121C, and the measurement uncertainty is ±7%. We performed a linear fit to the measured values, and the fictitious-field coefficient of the light-shift beam with a wavelength of 1083.195 nm was scaled to 3nT/mW according to the slope.

### 3.3. Response and Performance of Magnetometer

**Figure 4:**
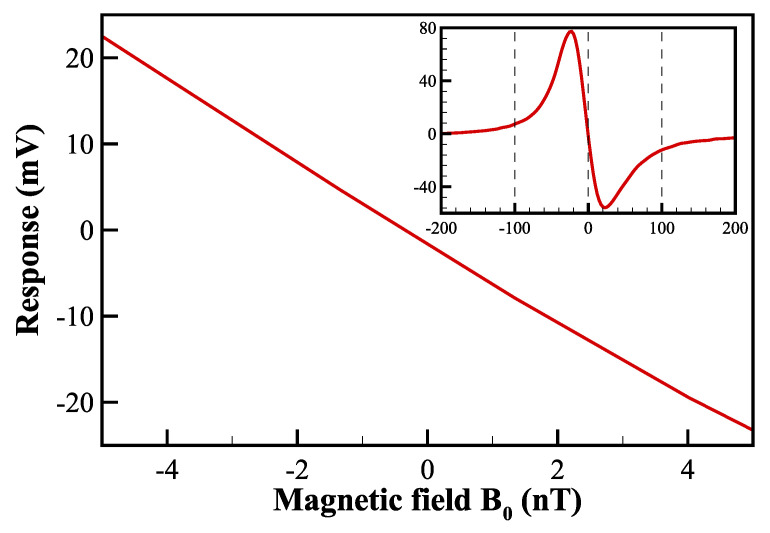
Response of the demodulated signal with respect to the magnetic field $B_{0}$. The inset shows the response to a wider range of magnetic fields ranging from −200 nT to 200 nT.

After the fictitious RF offset due to the non-zero mean intensity of the light-shift beam is canceled, the magnetic-field response, that is, the zero-field parametric-resonance signal is obtained by sweeping the $B_{0}$ field during demodulation, as shown in Figure 4. The amplitude of the optimized response is 130.7$\;{\mathrm{mV}}_{\mathrm{pp}}$, and the linewidth is 40.1 nT. In addition, the magnetic-field response is linear around the zero fields. Note that the response curve is not perfectly antisymmetric when observed over a large scanning range for three possible reasons. The first is the inhomogeneity of the fictitious-field distribution caused by the curved surface of the cylindrical gas cell on which the light-shift beam is incident, the second is the unwanted spurious pumping in the *z*-axis due to the residual absorption of the light-shift beam, and the third is the higher-order effects from the tensor light shift caused by the ${\hat{T}}_{0}^{\left(2\right)}$ term in Equation (2).

In open-loop measurements, the slope of the magnetic-field response in the linear region provides the relationship between the demodulated voltage and the $B_{0}$ field to be measured. The advantages of the open-loop operation are the simplicity of the required circuitry and the absence of electrical noise introduced by the servo loop. However, the slope of the magnetic-field response must be calibrated frequently and precisely to prevent inaccuracies in magnetic measurements. In closed-loop measurements, the magnetic field experienced by the ${}^{4}$He gas cell inside the magnetic shield is dynamically locked at zero fields by the third proportional-integral-derivative controller (${\mathrm{PID}}_{3}$ in Figure 2) according to the zero-crossing dispersive response. Note that the ${\mathrm{PID}}_{3}$ is a fully programmable controller embedded in the lock-in amplifier. The closed-loop error signal is injected into a feedback coil, which is used to generate a compensating field $B_{c}$ with the same magnitude and opposite direction as the $B_{0}$ field using the voltage-controlled current source (Stanford Research Systems CS580). By reading the compensating current on the feedback coil at 10 kHz sampling rate through National Instruments data acquisition devices controlled by LabVIEW software, the $B_{0}$ field is measured with a known coil constant. It should be noted that the parameters of ${\mathrm{PID}}_{3}$ are optimized by observing and analyzing the performance of the closed-loop noise spectral density in real time.

**Figure 5:**
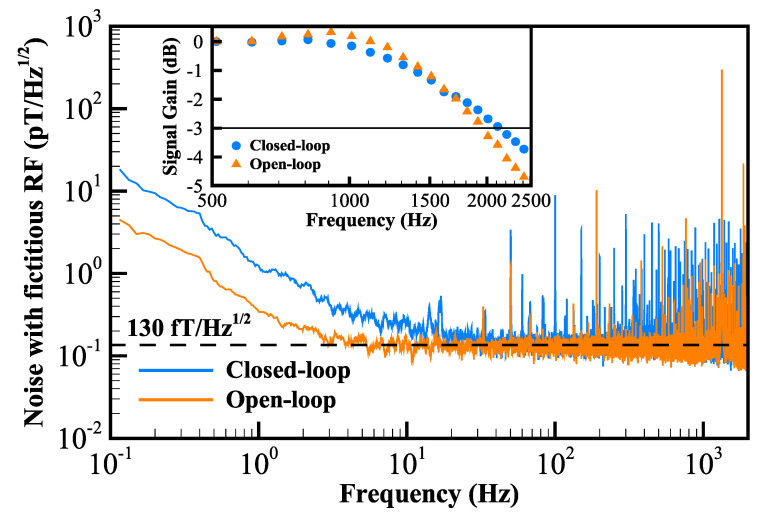
Spectral densities of the magnetic-field noise in open-loop (orange plot) and closed-loop (blue plot) operations of fictitious RF scheme. The sensitivities are approximately 130$\;\mathrm{fT}/{\mathrm{Hz}}^{1/2}$ for both modes. The closed-loop noise floor approaches that of the open-loop noise at frequencies above 40 Hz. The inset shows the signal frequency responses, demonstrating a measurement bandwidth of approximately 2 kHz.

Open- and closed-loop measurements were performed separately. Figure 5 shows the measured noise spectral densities. Both modes exhibit a magnetic-field noise floor of 130$\;\mathrm{fT}/{\mathrm{Hz}}^{1/2}$. Remarkably, in the low-frequency range, the sensitivity of the closed-loop mode is worse than that of the open-loop mode. We attribute the deterioration of the closed-loop sensitivity in the low-frequency range to two causes. One is the raised low-frequency electrical noise introduced by the voltage-controlled current source, and the other is the tradeoff between the proportional-integral-derivative parameters to suppress the noise in different frequency ranges. The closed-loop noise floor approaches that of the open-loop noise at frequencies above 40 Hz. In addition, the frequency spikes in the noise spectral densities are induced by the 50 Hz power frequency and the optical-intensity-modulation frequency of the light-shift beam. Considering the NMOR noise floor of 65$\;\mathrm{fT}/{\mathrm{Hz}}^{1/2}$ [41], MODR of 40$\;\mathrm{fT}/{\mathrm{Hz}}^{1/2}$ [42] and SERF of 2$\;\mathrm{pT}/{\mathrm{Hz}}^{1/2}$ [45] in pioneering works of optically pumped magnetometers based on all-optical design, the proposed all-optical PRM has a comparable sensitivity.

The inset of Figure 5 demonstrates the frequency responses of the open- and closed-loop measurements. Frequency responses were measured by applying an oscillating magnetic field of 12$\;{\mathrm{nT}}_{\mathrm{pp}}$ at different frequencies. The measured −3 dB bandwidths are 1.95 kHz for open-loop operation and 2.15 kHz for closed-loop operation, and both are approximately 2 kHz, which corresponds to the low-pass filter bandwidth of the lock-in amplifier.

## 4. Further Discussion

**Figure 6:**
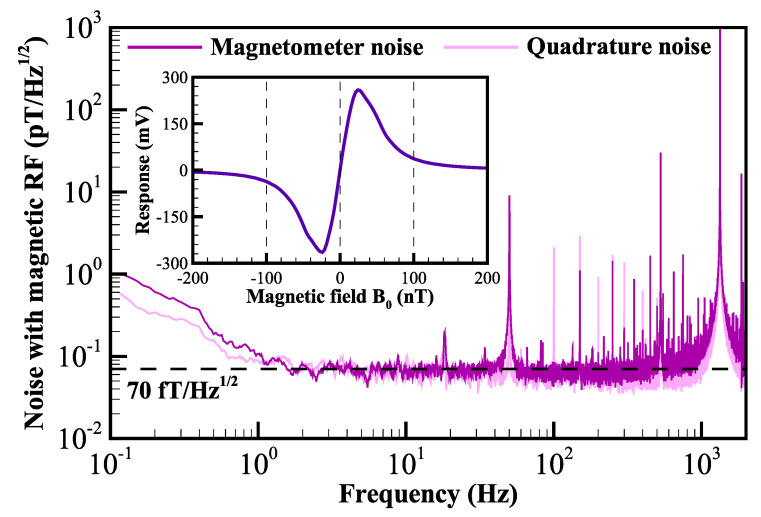
Spectral densities of the noise under the condition of using a magnetic RF field to induce parametric resonance. The sensitivity is approximately 70$\;\mathrm{fT}/{\mathrm{Hz}}^{1/2}$. The magnetometer noise floor is close to that of magnetic-insensitive quadrature-demodulated noise. The inset shows the magnetic response of the demodulated signal in the magnetic RF scheme.

To determine the potential for improved sensitivity, the magnetic RF alignment-based PRM was operated in the open-loop mode under the same apparatus and parameters, except for replacing the fictitious RF field with the magnetic RF field whose amplitude was optimized to 144$\;{\mathrm{nT}}_{\mathrm{pp}}$. The amplitude of the optimized response of the magnetic RF scheme is 519.6$\;{\mathrm{mV}}_{\mathrm{pp}}$, and the linewidth is 54.3 nT, as shown in the inset of Figure 6. The noise spectral densities when using a magnetic RF field show a sensitivity of 70$\;\mathrm{fT}/{\mathrm{Hz}}^{1/2}$, and the magnetometer noise floor is close to that of the magnetic-insensitive quadrature-demodulated noise (see Figure 6).

The difference in sensitivity between the fictitious RF scheme and the magnetic RF scheme is mainly due to the quality factor of the respective magnetic-field responses. Because the effective range of the fictitious RF field is only the irradiation area of the light-shift beam in the gas cell, which is smaller than that of the magnetic RF field covering the entire space of the gas cell, the amplitude-to-linewidth ratio of the magnetic RF scheme is three times that of the fictitious RF scheme. Furthermore, one can find that the signal linewidth of the fictitious RF scheme is about 10 nT smaller than that of the magnetic RF scheme because the magnetic gradient broadening caused by the fictitious RF field is probably smaller than that of the magnetic RF field. The effective range of the fictitious RF field is currently limited by the aperture of the magnetic shield and the optical power of the light-shift beam. Another factor that affects the sensitivity of the fictitious RF scheme is the optical noise introduced by the frequency jitter and drift of the light-shift beam, and this noise also brings measurement errors to the magnetometer. Therefore, in further optimization, we will consider the frequency stabilization of the detuned laser, such as dichroic atomic vapor laser lock and polarization-spectroscopy laser lock with a Doppler spectrum width. If these difficulties are addressed, the sensitivity of the fictitious RF scheme is expected to further reach 70$\;\mathrm{fT}/{\mathrm{Hz}}^{1/2}$ as is the case with the magnetic RF scheme. Compared with the magnetic RF scheme, the fictitious RF scheme has no RF crosstalk and is of benefit to array-based biomagnetic applications, such as magnetocardiography and magnetoencephalography.

In the future, the frequency or polarization modulation of the light-shift beam will be designed to generate a null-offset fictitious RF field for all-optical parametric resonance. In addition, the second light-shift beam can be applied along the *y*-axis to achieve three-axis measurements.

## 5. Conclusions

In summary, this paper has demonstrated an optically modulated alignment-based ${}^{4}$He atomic PRM, which provides an alternative method to induce alignment-based parametric resonance. The fictitious RF field generated by the modulated light shift is used to realize the parametric resonance to eliminate the crosstalk caused by the magnetic RF field. The relative intensity noise of the lasers is suppressed to optimize the measurement sensitivity. The proposed magnetometer experimentally shows a sensitivity of 130$\;\mathrm{fT}/{\mathrm{Hz}}^{1/2}$ in both open- and closed-loop operations and is considered to have the potential to reach 70$\;\mathrm{fT}/{\mathrm{Hz}}^{1/2}$ when compared with the magnetic RF scheme operated under the same geometry. Our PRM provides near-zero magnetic-field measurements with a 2 kHz bandwidth at room temperature, which would be useful for high-bandwidth measurements in biomagnetic applications. 

## Data Availability

The data that support the findings of this study are available from the corresponding author, upon reasonable request.
